# Fabrication, Characterization, and Biological Activity of Avermectin Nano-delivery Systems with Different Particle Sizes

**DOI:** 10.1186/s11671-017-2405-1

**Published:** 2018-01-09

**Authors:** Anqi Wang, Yan Wang, Changjiao Sun, Chunxin Wang, Bo Cui, Xiang Zhao, Zhanghua Zeng, Junwei Yao, Dongsheng Yang, Guoqiang Liu, Haixin Cui

**Affiliations:** 1 Institute of Environment and Sustainable Development in Agriculture, Chinese Academy of Agricultural Sciences, Beijing, People’s Republic of China; 2 Nanobiotechnology Research Center, Chinese Academy of Agricultural Sciences, Beijing, People’s Republic of China

**Keywords:** Avermectin, Nano-delivery system, Controlled release, Biological activity

## Abstract

Nano-delivery systems for the active ingredients of pesticides can improve the utilization rates of pesticides and prolong their control effects. This is due to the nanocarrier envelope and controlled release function. However, particles containing active ingredients in controlled release pesticide formulations are generally large and have wide size distributions. There have been limited studies about the effect of particle size on the controlled release properties and biological activities of pesticide delivery systems. In the current study, avermectin (Av) nano-delivery systems were constructed with different particle sizes and their performances were evaluated. The Av release rate in the nano-delivery system could be effectively controlled by changing the particle size. The biological activity increased with decreasing particle size. These results suggest that Av nano-delivery systems can significantly improve the controllable release, photostability, and biological activity, which will improve efficiency and reduce pesticide residues.

## Background

Pesticides are important for controlling plant diseases and insect pests and to safeguard national food security. Most conventional pesticide formulations are open systems, which have problems such as poor dispersion, degradation of active ingredients, and droplet drift. Active ingredients in pesticides have loss rates of up to 70–90%. This occurs as a consequence of field spraying owing to biodegradation, chemical degradation, photolysis, evaporation, surface runoff, and percolating ground water, all of which raise concerns for food safety and the environment [1, 2]. Improving the application and delivery of pesticides has therefore become an important research topic [3–5].

In recent years, the development of nanotechnology and nanomaterials has provided a new approach for improving the efficiency of pesticide application [6–15]. Nanoparticle formulations of pesticides have been proposed to produce a better spatial distribution of pesticides on leaf surfaces, owing to nanoparticles’ small size and large surface area, which provides better efficiency [16–19]. Nano-delivery systems for pesticides involve entrapping the active ingredients of pesticides inside polymeric nanomaterials, to allow the slow and controlled release of active ingredients on target crops [20–22]. Among different polymers, polylactic acid (PLA) has been extensively used as nanoparticle carriers in controlled release nano-delivery systems for many bioactive molecules due to its non-toxic, good bioavailability and biocompatibility, and approval by the Food and Drug Administration for human use [23, 24]. There are limited study about PLA as carrier materials in the field of pesticide. PLA is a very promising coating material to encapsulate pesticide due to its environmental friendliness, low cost, and easy scale-up.

Nano-delivery systems for pesticides also provide enlarged contact areas among the target pest and pesticide particles [25, 26].

Avermectin (Av) is a highly efficient, broad-spectrum, and safe biopesticide that can control a variety of agricultural pests. Av degrades easily owing to photo oxidation. Av also has a short half-life in water, which affects pest control in field applications. The coefficient of organic carbon adsorption for Av is high. This means that Av does not readily migrate in soil because it tightly binds with organic matter, which affects its pest control in soil. Much effort has focused on protecting the biological activity of Av by microencapsulation technologies [27, 28]. However, the size of Av-containing microcapsules is difficult to control. They are generally large, with sizes of approximately 1–5 μm, and have wide size distributions [29, 30]. Poor dispersion and uniformity, as well as large sizes, are not conducive to improving pesticide adhesion on the surface of leaves or increasing the permeability in harmful insects. Limited investigations have been conducted on the synthesis and biological activity of pesticide nano-delivery systems for Av with different sizes [31–34]. Constructing nano-delivery systems for Av by nanomaterial encapsulation can significantly improve its photostability, reduce its soil adsorption or other adverse factors, and improve the control effect of the pesticide. In addition, Av nano-delivery systems also have better penetration and allow for slower and more controlled release of active ingredients on target crops, compared with conventional microcapsules.

The present study aimed to prepare various particle sizes of Av nano-delivery systems by emulsion polymerization using PLA and characterize their performance as a safe and biodegradable carrier. We investigated the effect of particle size on the release properties and biological activity of the Av nano-delivery system [35–39]. The concentrations of the active substance and its precursors, and the characteristics of the emulsion system are the primary factors for establishing the size distribution of the final Av nano-delivery system. The Av nano-delivery system showed good particle dispersion with controlled particle size, high Av loading, effective size-control and sustained release properties, and good ultraviolet (UV) shielding and stability.

## Experimental

### Materials

PLA and Av were provided by Nature Works, and Qilu Pharmaceutical Co., Ltd. (Inner Mongolia, P. R. China), respectively. Polyvinyl alcohol (PVA), 87–90% hydrolyzed with an average M_w_ of 30,000–70,000, was purchased from Sigma-Aldrich Shanghai Trading Co., Ltd. (Shanghai, People’s Republic of China). Gelatin was purchased from Sinopharm Chemical Reagent Co., Ltd. (Beijing, People’s Republic of China). The dialysis membranes were purchased from Beijing Tianan Technology Co., Ltd. (People’s Republic of China). Other chemical reagents were of analytical grade and were purchased from Beijing Chemical Works (Beijing, People’s Republic of China). Water used in all experiments was of Milli-Q grade (18.2 MΩ cm, TOC ≤ 4 ppb) and was obtained from a Milli-Q Advantage A10 system (Millipore, Milford, MA, USA).

### Preparation of Avermectin Nano-delivery System

The Av nano-delivery system was prepared via an oil-in-water (O/W) emulsion method combined with an ultrasonic and shearing physical emulsification process. Briefly, PLA and Av were dissolved in methylene chloride as the oil phase. For the water phase, gelatin was dissolved in water at 40 °C, which was then mixed with PVA aqueous solution. Then, the oil phase was dripped slowly into a large volume of the water phase under high shear emulsification (FA25, FLUKO, Ruhr-gebiet, Germany), to prepare a coarse emulsion. The coarse emulsion was then uniformly dispersed by ultrasonic emulsification (JY 92-IIN, SCIENTZ, Ningbo, People’s Republic of China). The uniform emulsion was then solidified under magnetic stirring overnight (RW20, IKA, Staufen, Germany). The hardened Av nano-delivery system was collected via centrifugation and was washed three times with deionized water. Products were collected by centrifugation and then freeze-dried (FD-81, EYELA, Tokyo, Japan) to yield a free-flowing powder. The dried powder was stored at 4 °C until use.

### Characterization of Nano-delivery Systems

The morphology of each Av nano-delivery system was investigated by scanning electron microscopy (SEM, JSM-6700 F, JEOL Ltd., Akishima-shi, Japan) with an accelerating voltage of 5 kV. SEM samples were deposited dropwise onto the surface of a silicon slice. The droplet was allowed to dry at room temperature and was then coated with a thin layer of platinum using a sputter coater (EM ACE600, Leica, Vienna, Austria), to prevent charging during SEM observation. The sizes of particles in the Av nano-delivery systems were measured at 25 °C by laser scatter using a zetasizer (Zetasizer NanoZS90; Malvern, Worcestershire, UK).

### Determination of Avermectin Loading in the Nano-delivery Systems

The amount of Av in the nano-delivery system was measured at a wavelength of 245 nm, using an ultraviolet-visible (UV-vis) spectrophotometer (TU901, Shimadzu Corporation, Kyoto, Japan). In detail, the Av-loaded specimens were weighed out and dissolved in chloroform overnight, after which the solution was dried via reduced-pressure distillation. Methanol was then added to dissolve the Av from the dried precipitate. Finally, the mixture was filtered to yield a clear solution that was analyzed by UV-vis spectrophotometry.

### Controlled Release of Avermectin from the Nano-delivery Systems

The release profiles of Av from the nano-delivery systems of different sizes were investigated as follows. Av nano-delivery samples of each size were suspended in 10 mL of ethanol/water mixture (1:1, *v*/*v*). The suspension was then transferred to a dialysis bag, which was sealed in a brown flask with 90 mL of ethanol/water mixture (1:1, *v*/*v*) as the release medium. The flask was incubated in an incubator shaker at 300 rpm at room temperature. After defined time intervals, 5.0 mL of solution was removed and replaced with 5.0 mL of fresh solvent. The release rate of Av from the nano-delivery sample was calculated by measuring the concentrations of Av dissolved in the release medium at different intervals and was used to evaluate the sustained release property. The concentration of Av was measured using a UV-vis spectrophotometer at a wavelength of 245 nm. Technical abamectin (TC, technical grade active ingredient) was used as controls.

### Photolysis Behavior of Avermectin in the Nano-delivery System

The photolytic behavior of Av in the nano-delivery system was evaluated with the commercial Av WDG as a control. The samples were dissolved in methanol/water (1:1, *v*/*v*) and divided equally into culture dishes, and the resulting samples were irradiated for a desired duration at 25 °C under an UV lamp (500 W), which had a maximum intensity at a wavelength of 365 nm. At specified time intervals (12, 24, 36, 48, 60, and 72 h), the culture dish was taken out of the reactor and the Av concentration of samples was analyzed.

### Stability Tests

The stability of the Av nano-delivery system was tested according to CIPAC MT 46 and GB/T 19136–2003. Samples were packed in glass tubes and stored at 0 ± 2 °C for 7 days and 54 ± 2 °C for 14 days. Changes in the amount of Av in the nano-delivery system were then studied.

### Bioassays

Bioassays of the Av nano-delivery system of different sizes were conducted using the leaf-dip method. Samples were diluted with Triton X-100 aqueous solution at different Av concentrations. Cabbage (*Brassica oleracea* L.) leaves were immersed in the diluted Av suspension, then dried at room temperature, and attached to a Petri dish. Aphid larvae were introduced into each dish, and the treated aphids were cultured in an incubator at 25 °C and 75% relative humidity. Four replications were conducted to compare with the control test. Mortality was assessed at 48 h after treatment. Concentration-mortality data were analyzed using DPS v12.01 statistical software. The median lethal concentrations (LC_50_) and their 95% confidence limits were calculated. Commercial WDG was used as a control.

## Results and Discussion

### Construction and Characterization of the Avermectin Nano-delivery System

**Fig. 1:**
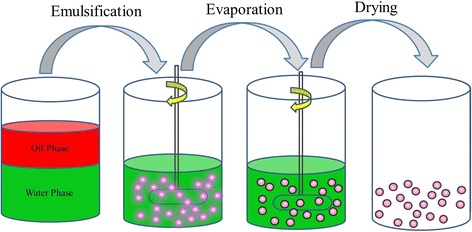
Schematic showing the preparation of the Av nano-delivery system

**Fig. 2:**
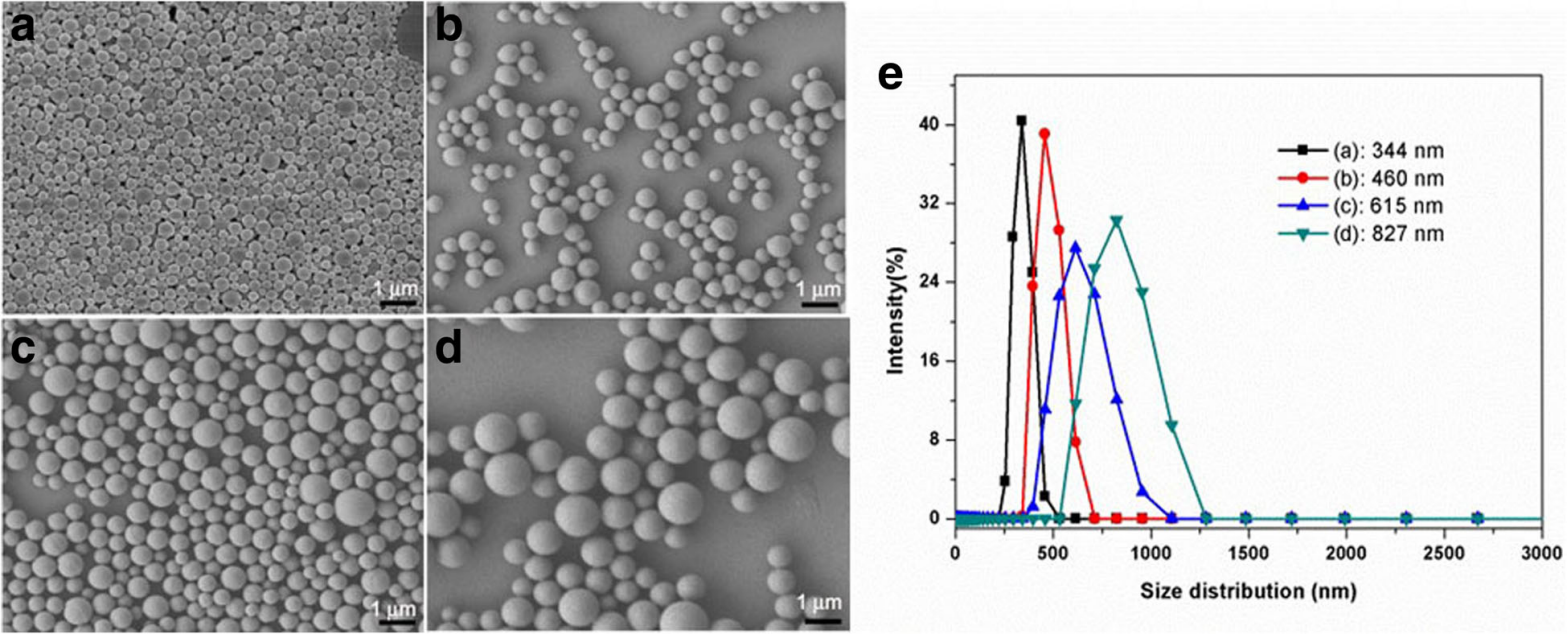
SEM images (**a**–**d**) and size distributions (**e**) of Av nano-delivery systems with different particle sizes

**Fig. 3:**
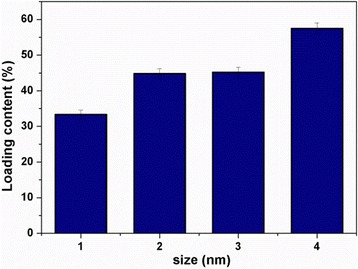
Amounts of Av in Av nano-delivery systems with different particle sizes

The Av nano-delivery systems were constructed according to the procedure shown in Fig. 1. During the process, the water and oil phases were prepared according to the method mentioned above. The oil phase was emulsified in a water phase (oil-in-water emulsion) by high shear emulsification, to prepare a coarse emulsion. The course emulsion was then uniformly dispersed by ultrasonic emulsification. This was followed by stirring, evaporation, and centrifugation of the resulting nanoparticles. The particle size of a delivery system is one of the most important factors affecting the release properties and biological activity of the pesticide. As shown in Fig. 2, Av nano-delivery systems with particle sizes ranging from 344 to 827 nm were constructed by controlling the synthesis parameters. Particle size is an important parameter for the controlled release properties of pesticides. The particle size of the Av nano-delivery systems could be controlled by changing the PVA/gelatin concentration ratio. Various Av nano-delivery systems were prepared with sizes ranging from 344 to 827 nm and Av contents ranging from 33.4 to 57.5% (33.4, 44.9, 45.2, and 57.5%), as shown in Fig. 3. All the Av products had smooth surfaces and spherical particle morphologies.

### Avermectin Release from the Nano-delivery System In Vitro

**Fig. 4:**
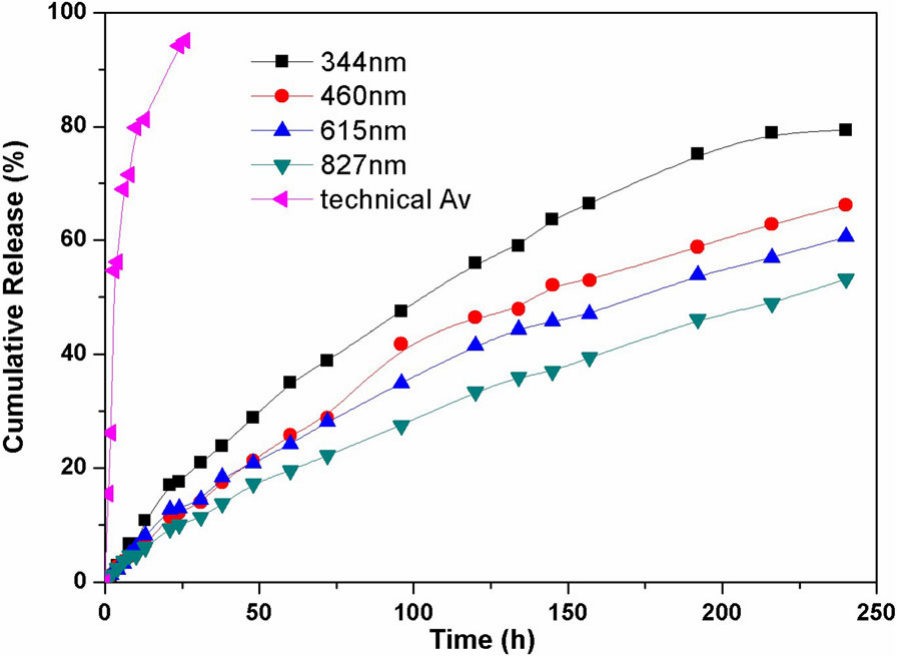
Release behaviors of Av nano-delivery systems with different particle sizes in ethanol/water (50:50, *v*/*v*) over 200 h

In recent years, the development of pesticide-release systems has transitioned toward accurate and quantitative release, in contrast to earlier slow and qualitative release systems. To achieve controllable release, the release profiles of the Av nano-delivery systems with various particle sizes were systematically investigated. Figure 4 shows the percentage release of Av from the nano-delivery systems with different particle sizes after the same time interval. The technical Av had a fast release rate and was almost completely released after 25 h. Pesticide lasting validity period needs the sustained release of pesticides to maintain efficacy for a long time. Compared to burst release of the technical abamectin, all the prepared nano-delivery systems released Av at relatively slow speeds and maintained sustained release for longer periods. Av release profiles from the nano-delivery systems consisted of a burst release followed by a gradual release over the 240-h time frame of the experiment. As the delivery system size decreased from 827 to 344 nm, the cumulative release increased from 53.2 to 79.4% after 240 h. The results indicated that the Av release rate from the nano-delivery system gradually increased with decreasing particle size. This was due to a higher surface area being exposed to the surroundings, aiding permeation and effusion of pesticide located in the shells of the nano-delivery system. The results showed that the Av release rate from the nano-delivery system could be effectively controlled by modifying the particle size.

### Biological Activity

**Fig. 5:**
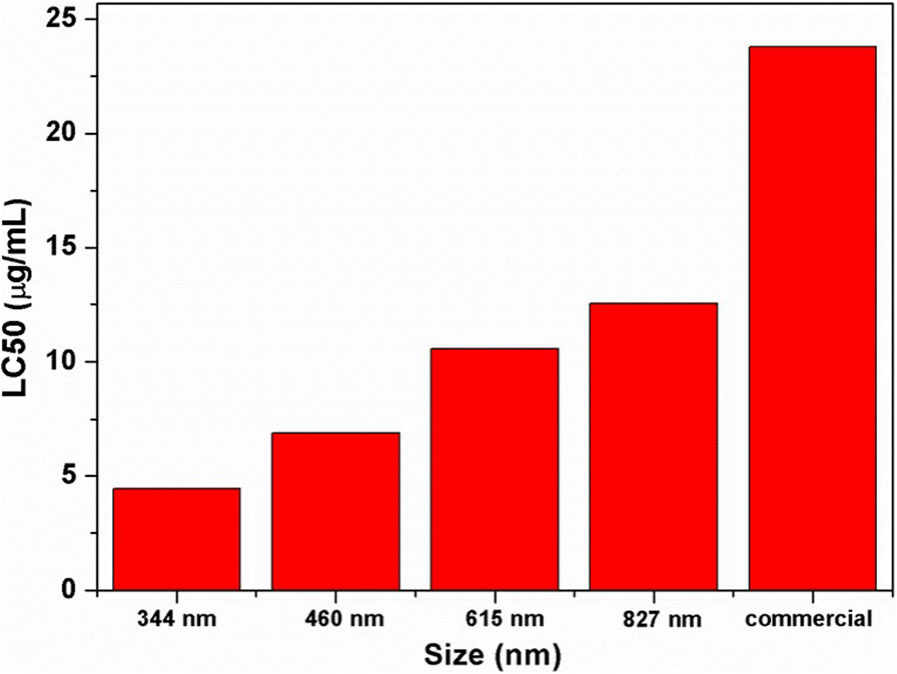
Bioassay results of Av nano-delivery systems with different particle sizes

The biological activity of Av released from nano-delivery systems of different particle sizes against aphids is shown in Fig. 5. The LC_50_ of the Av nano-delivery system gradually decreased with decreasing particle size. The bioavailability of nanoemulsions is reportedly higher than that of conventional emulsions because of their smaller particle size and higher surface-to-volume ratio. Therefore, the higher biological activities of Av nano-delivery systems with smaller particle sizes were attributed to the increased dispersibility, wettability, and retention caused by small-scale effects. All the Av nano-delivery systems had lower LC_50_ values and higher activities than commercial Av WDG. The high efficacy was due to the nano-sized particles enhancing adhesion and penetration of the Av pesticide on the surface of crops, which reduces loss of pesticide due to leakage during spraying.

### UV-Shielding Properties of Avermectin in the Nano-delivery System

**Fig. 6:**
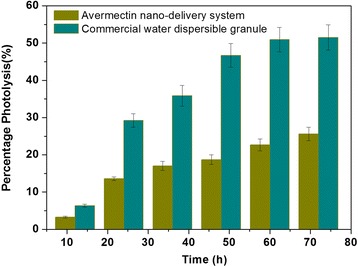
Comparison of Av photolysis percentage with the commercial WDG and nano-delivery system under UV irradiation

To verify the UV-shielding properties of Av in the nano-delivery system, the photolytic rate of Av was estimated by artificial irradiation. The analysis of the photolysis rate of Av with irradiation time is shown in Fig. 6. The photolytic percentage of abamectin was 18.7% for the nano-delivery system and 46.7% for the commercial Av WDG after 48 h. After 72 h, the photolytic percentage of abamectin was 25.6% for the nano-delivery system and 51.5% for the commercial Av WDG. These results showed that the nano-delivery system exhibited inhibited photolysis of Av because of the protective effect of the wall carrier.

### Storage Stability

**Fig. 7:**
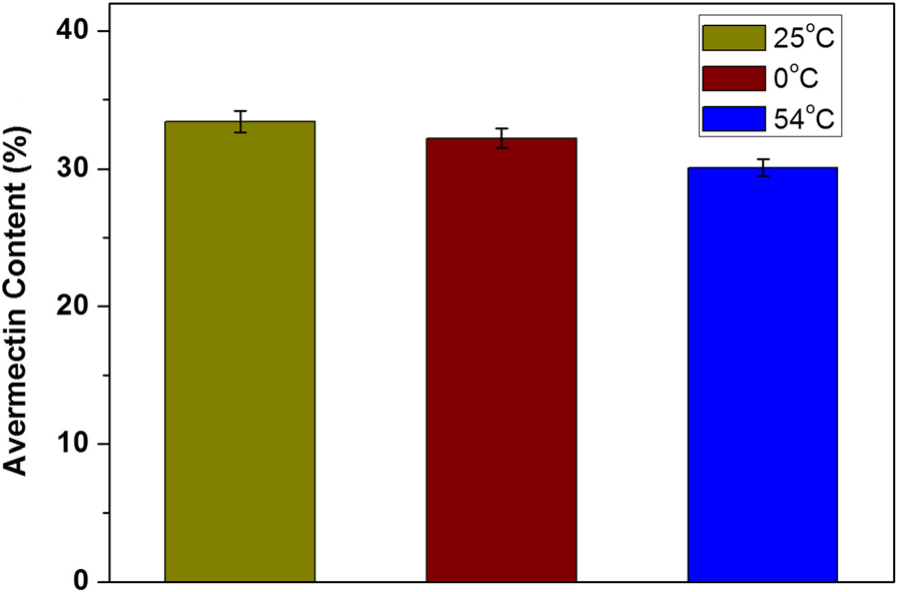
Stability of Av nano-delivery system at different storage temperatures

The stabilities of the Av nano-delivery systems with different particle sizes were evaluated by measuring their loading contents at temperatures of 0, 25, and 54 °C. Figure 7 shows that the nano-delivery system remained stable with no major changes in Av loading during storage at room temperature and low temperature. A small loss of Av was observed after 14 days at 54 °C, owing to the degradation of Av at high temperature. These results showed that the Av nano-delivery system had good storage stability.

## Conclusions

To improve the controlled release, chemical stability, and bioactivity of Av, an Av nano-delivery system with different average particle sizes was synthesized using the emulsion polymerization method. The Av nano-delivery system showed consistent release behavior. The Av release rate from the nano-delivery system gradually increased with decreasing particle size, owing to higher surface area. The biological activity of the Av nano-delivery system gradually increased with decreasing particle size, owing to enhanced adhesion and penetration. The Av nano-delivery system showed good anti-photolysis properties and stability. The delivery system overcomes the shortcomings of current biopesticides, such as their environmental sensitivity, undesirable soil adsorption, and short activity duration. This will improve pesticide efficacy and decrease the required spraying frequency.
